# A novel use for an old drug

**Published:** 2008

**Authors:** Sonia M.D. Brucki

Dimebon has been used in Russia as a non-selective antihistamine for many years. The drug seems to act on mitochondria and inhibit neuronal death, weakly inhibiting butyrylcholinesterase and acetylcholinesterase and weakly blocking NMDA receptors.

In this recently published trial Dimebon was given to two groups of mild to moderate Alzheimer’s disease patients (n=183) at 11 sites in Russia, randomized for 60 mg/day of dimebon or placebo, for 26 weeks. Patients were not taking other drugs, such as anticholinesterasics or NMDA antagonists.

The study was completed by 155 patients. The primary outcome endpoint considered was score on ADAS-Cog, with a significant positive difference seen in scores for dimebon (mean improvement of 2 points) whereas the placebo group decreased more than 2 points. All other secondary outcome measures were significantly better in the dimebon group (MMSE scores; NPI – Neuropsychiatric Inventory – measure of behaviour; ADCS-ADL – Alzheimer’s disease Cooperative Study – activities of daily living; Clinician’s Interview-based Impression of Change plus Caregiver Input – CIBIC-plus). An extension of the study was conducted for 134 patients to 52 weeks, with continued better outcome on all five measures. The outcome profile of dimebon was similar to cholinesterase inhibitors. Dimebon was very safe, with dry mouth occurring more frequently in the dimebon group. Overall frequencies of adverse events were quite low.

This trial should be replicated in other countries and for a greater number of patients. In this trial only one single dose dimebon was studied, therefore future studies should be performed to verify efficacy with other dose regimens.

We view Dimebon as a promising drug, particularly after studying dimebon associated to anticholinesterasics drugs and/or with memantine.

**Sonia M.D. Brucki**
